# Common or multiple futures for end of life care around the world? Ideas from the ‘waiting room of history’

**DOI:** 10.1016/j.socscimed.2016.11.012

**Published:** 2017-01

**Authors:** Shahaduz Zaman, Hamilton Inbadas, Alexander Whitelaw, David Clark

**Affiliations:** School of Interdisciplinary Studies, The University of Glasgow, Crichton University Campus, Dumfries, DG1 4ZL, Scotland, UK

**Keywords:** End of life, Global palliative care, Global health, Quality of death, Death and dying, Waiting room of history, Post-colonial theory

## Abstract

Around the world there is growing interest in the manner in which care is delivered to people at the end of life. However, there is little unanimity on what constitutes a ‘good death’ and the appropriate societal responses to the issue of delivering culturally relevant and sustainable forms of end of life care in different settings are not subjects of broad agreement. In this critical conceptual paper we focus on the emerging narratives of global palliative care and offer an assessment of their implications. We relate this to calls to improve end of life care across jurisdictions and settings, attempts to map and grade the development of palliative care provision, and to the emergence of a widely recognised global ‘quality of death index’. We consider an alternative approach to framing this debate, drawn from a subaltern and post-colonial studies perspective and suggest that adopting a truly global perspective will require acceptance of the plurality of past and present local problems and issues relating to end of life care, as well as the plural possibilities of how they might be overcome. In that context, we would not aim to universalise or privilege one particular global future for end of life care. Instead of homogenising end of life interventions, we seek to be open to multiple futures for the care of the dying.

## Highlights

• Post-colonial perspective re-frames debate on the future of global palliative care.
• Provides innovative critique of global rankings of ‘quality of death’.
• Advocates multiple futures for end of life care in different settings.
• Applies much needed theoretical approach to debates on global end of life care.

## 1 Introduction

Global interest in end of life care is growing, for compelling demographic and epidemiological reasons (Cohen and Deliens, 2012). The population of the world is ageing and increasing. The number of people dying each year is set to rise. For many the process of dying will become more extended, as life threatening diseases transform into chronic conditions and the idea of a ‘terminal’ illness may mean death within years, rather than months, weeks or days. For others death may still come quickly – from new infectious diseases, natural disaster, and human made catastrophes of many kinds including war, mass migration, poverty and famine. There are many complexities in the challenge of providing appropriate care at the end of life across so many unique circumstances and contexts.

Yet how we die, what constitutes a ‘good death’ and the appropriate societal responses to the issue of delivering culturally relevant and sustainable forms of end of life care in different settings are not subjects of broad agreement. Rather, they can be seen as a ‘contested space’, where ideas, policies, practices and professions compete to define clear solutions. Decades back Rittel and Webber (1973) argued that in a pluralistic society there is nothing that constitutes the undisputable public good and it makes no sense to talk about ‘optimal solutions’. Yet the work of the modern hospice and palliative care movement seems often to be characterised in this light – as something self-evidently beneficial to all, indeed something that constitutes a human right. In this paper we examine the emerging narratives of global palliative care and offer a critical assessment of their implications. We relate this to calls to improve end of life care across jurisdictions and settings, attempts to map and grade the development of palliative care provision, and to the emergence of a widely recognised global ‘quality of death index’. We consider an alternative approach to framing these issues, drawn from a subaltern and post-colonial studies perspective. We conclude with some reflections on how the ‘field’ might more realistically tackle the problem of making appropriate end of life care available to all who seek it.

Our work is based on a close and critical reading of published texts, papers, commentaries and reports. We also make use of a published interview and memoir in which two palliative care activists set out their experiences and views.

### 1.1 Global disparities in palliative care

The global history and development of hospice and palliative care has recently been described in depth (Clark, 2016). The nineteenth-century saw major demographic and social changes in western countries that began to transform how people died. As lives lengthened, so the manner of their ending was transformed. The modernist shift in the construction of dying from a religious process to a medical one was getting underway. New modes of pain relief were brought on by the isolation of morphine and the invention of the hypodermic syringe. Medical texts began to give greater attention to the last phases of life and the medical management of the sick room when death is near (Munk, 1887). From the late nineteenth and early twentieth century, special terminal care homes and hospices were established in London, New York and several European cities as well as one Indian city (Pondicherry). Usually religiously inspired and small scale, they developed a particular philosophy of care which later motivated others, from the middle of the twentieth century onwards. Indeed some of the homes survived as institutions and made the transition into the world of modern hospice and palliative care.

By the mid-twentieth century medical, nursing and social work commentary on the care of the dying began to increase. There was a growing concern about the medical neglect of dying people, the problems associated with the ‘futile’ treatment of advanced disease, and the influences of a wider ‘death denying’ culture that made end of life issues difficult to address, both socially and culturally as well as medically.

This began to change in western countries such as Britain and the United States from the early 1960s. This was first manifested in a body of new writing on the subject and then in the establishment of new services at the community level, which coalesced around modern ‘hospice’ principles. These new settings not only provided specialist care for those close to death, but also began to promulgate associated teaching and research. The work of Cicely Saunders and her associates was key to this (Saunders, 1966), but social science critiques of care at the end of life also played their part (Glaser and Strauss, 1965, Glaser and Strauss, 1968). The new approaches sought to promote dignity at the end of life, to address problems of pain and other symptoms, to encourage the involvement of different disciplines, including volunteers, and to acknowledge that suffering has several dimensions – physical, social, psychological and spiritual (Saunders, 1964).

By the mid-1970s this orientation was taking on the name ‘palliative care’ (Overy and Tansey, 2013). Quite quickly it found advocates within the health care system, building on the achievements of the hospice founders who had been largely oriented to endeavours outside of the mainstream in free-standing charitable, non-government and non-profit organisations. It also began to gain interest in many countries around the world. Originally, the focus of this work was on those dying from cancer, but this soon began to change. The benefits of palliative care for people with other non-communicable conditions came to be recognised and in due course life threatening infectious disease also attracted the attention of palliative care specialists. In some quarters there was also a desire to move the initial point of intervention further back in the disease trajectory so that ‘early’ palliative care could be offered, rather than introducing palliative care only in the very final stages of life. In due course, as we shall see, this led to numerous definitional and terminological debates within the field. Despite these, palliative care continues broadly to be associated with care when death is approaching and if palliative care is not necessarily *synonymous* with end of life care; the former is certainly a part of the latter. There is also a growing distinction between ‘specialist’ and ‘generalist’ palliative care (Quill and Abernethy, 2013). The former is seen as the preserve of those who use advanced skills and conduct research and education focussed exclusively on people with palliative care needs, often in settings dedicated to this purpose. The latter is seen as an array of skills that can be practised in a variety of settings where those with palliative care needs make up only a portion of those being served. Within the literature it is often unclear which of these is being described and it is easier to track the development of the first than the second.

Another critical feature in this trajectory has been the linkage between palliative care and public health. From the early 1980s the World Health Organization (WHO) turned attention to the global problem of cancer pain relief and then to the wider issue of palliative care. Seeking what would now be called scalable solutions to these issues, WHO began to use the language of public health to define and endorse the principles of palliative care in the global context (Clark, 2016).

The WHO estimates that there were approximately 54.6 million deaths worldwide in 2011 and that over 20 million people every year could benefit from palliative care at the end of life (WHO, 2014). The majority of these (69%) are adults over 60 years old and some 6% are children. The highest proportion (78%) of adults who could benefit from palliative care at the end of life are living in low and middle-income countries, but the most developed levels of palliative care provision are found in the higher-income countries. Those dying from non-communicable diseases represent around 90% of the burden of end of life palliative care. The top conditions are cardiovascular diseases, cancer, and chronic obstructive pulmonary diseases. The vast majority (98%) of children in need of palliative care at the end of life also belong to low and middle-income countries, and within this group 83% are in the lower income categories, where the highest need is found. In addition, the rise of infectious diseases such as HIV/AIDS and multidrug-resistant tuberculosis, as well as complex humanitarian emergencies occurring in many developing countries also cause suffering and require pain control and palliative care for patients and support for their families – opening up new areas for palliative care intervention (Knaul et al., 2015).

Despite the number of people dying in low and middle-income settings, very little is known from a research perspective about how palliative and end of life care are being structured and delivered in developing countries. One systematic review showed that 90% of palliative care studies focus on just a few specific European countries (Pastrana et al., 2010). Another indicates that most international palliative care research (involving two or more countries) is taking place in high income settings (Clark et al., 2016). We note the English language limitations of this literature, but consider it unlikely that much relevant work has been published in other languages. With only a few exceptions (the Nordic countries, Romania, Spain, Turkey) specialist end of life care and thanatological journals are published in English.

A gross inequity between developed and developing countries can be found in access to pain control. High-income countries account for nearly 92% of medical morphine consumed in the world, but comprise only 17% of the total population. In contrast, low- and middle-income countries, representing the remaining 83% of the world's population, account for a mere 8% of the total morphine consumption (INCB, 2011). Whilst the consumption of opioids for medical purposes is increasing in North America, Western and Central Europe and Oceania, everywhere else this is not the case (Berterame et al., 2016).

Likewise, global mapping of the levels of palliative care development shows wide variations with regard to the preparedness of health systems for palliative care in developed and developing countries (Lynch et al., 2013). The situation varies from informal family-based palliative care alone, to isolated clinical care service levels, to the ‘advanced integration’ of palliative care within mainstream health systems, albeit with only 22 countries in this latter category. In the vast majority of developing countries, there are only meagre levels of institutional palliative care and there is limited or no evidence of its recognition in health policy and a scarcity of specifically trained personnel to support it (Gysels et al., 2011, Bingley and Clark, 2009, McDermott et al., 2008, Callaway et al., 2007). The overall picture is one of striking variation in palliative care provision across the globe (Clark, 2010).

These analyses are sometimes confounded by definitional issues. There has been significant debate—and no lack of disagreement—about the various definitions and models of palliative, end of life, and hospice care that now exist. The WHO has produced two definitions of palliative care (World Health Organisation, 1990, Sepúlveda et al., 2002) but many more are described in the literature. These definitional problems continue to inhibit clarity of thought and action in the field. For example, a paper focusing on definitions of the term *palliative medicine* and *palliative care* in two languages found a total of thirty-seven English and twenty-six German versions, confirming a lack of a consistent meaning about key terms and approaches (Pastrana et al., 2008). One American study tested a ‘new language’ definition of palliative care and suggested it should be used when defining or describing palliative care for consumers, seeing palliative care as ‘specialized medical care for people with serious illnesses’ and not limited to care at the end of life (Center to Advance Palliative Care, 2011).

In this paper we acknowledge these unresolved debates. We take the view that it is not possible to make a settled distinction between, for example, ‘palliative care’ and ‘palliative medicine’. The latter is often used to refer to the broader field as a synonym for palliative care, or it can refer specifically to the purely medical field of specialization that is now recognized in over 20 countries of the world. The two usages are not always clearly explained.

Added to this are the enormous cultural disparities, varied meanings and understandings of dying and death, which in turn shape perceptions and the practice of palliative care. It is helpful to see palliative care as an intersubjective process involving a number of stakeholders such as formal care providers, patients and their family members. The participation of these actors will play out differently in varied and sometimes conflicting (sub) cultures, based on their own priorities. The body of the dying person can thus become an arena for contested cultural assumptions. This can become further complicated where there is diversity in the cultures of ‘clients’ and ‘providers’ – a context that becomes more common in a globalising world (Gysels et al., 2012a, Gysels et al., 2012b, Clark, 2012; Crippen, 2008; Gebara and Tashjian, 2006).

Apart from the culture of role based groups, there are cultures of communities in particular geographical contexts. Cultural differences are particularly in evidence in end of life situations where there are specific ideas about what a ‘good death’ entails. Social scientists and clinicians have offered varying definitions and perspectives on ‘the good death’. The concept can unify around the ideal of dying with dignity, peacefulness, preparedness, awareness, adjustment and acceptance (Hart et al., 1998). Walter (2003) takes the view that the good death depends explicitly on the social context and specifically on the degree of secularization, of individualism and the length of time it takes to die (‘quick’ or ‘slow’). It is widely acknowledged that hospice and palliative care might become a form of institutionalised ‘good death’ (McNamara et al., 1994, McNamara, 2004). One review of 36 studies where participants included patients, family members, and healthcare providers identified 11 core themes of good death of which the top three were: preferences about the dying process (94% of reports), pain-free status (81%), and emotional well-being (64%) (Meier et al., 2016).

Seen more generally, some key areas of disparity between Western and non-Western countries are found in views about: the appropriate *place* where death should occur; *religious and spiritual expectations* surrounding death; and laws and opinions about *euthanasia or assisted dying*. While most deaths in the non-Western context take place in the home, in Western countries deaths increasingly take place in institutions. End of life care in Western hospices has been seen as an example of the sequestration of death from social life (Lawton, 1998). However, a scoping exercise in seven European countries reflects clearly distinguishable national cultures of end of life care, with differences in meaning, priorities, and expertise in each country (Gysels et al., 2012a, Gysels et al., 2012b, Gysels et al., 2012c). In general a high value is given to autonomy, the right to information, freedom of choice and the dignity of a dying patient in the Western context. However, the situation is different for non-Western migrants living in a Western country. Gunaratnam (2013) for example in her study on transnational dying and care in British cities shows how for migrants, ‘belonging’ and ‘home’ become crucial when making sense of death in a foreign land.

In contrast to Western countries, different concerns and practices around death and dying are revealed in various other contexts. For example, a systemic review of end of life care in Sub Saharan Africa shows that palliative care is provided essentially at home and by informal carers including women, the elderly, as well as children (Gysels et al., 2011). In Ghana, an emphasis is found on the burial, rather than the dying person. Here the sick bed and the process of dying tend to be confined to the seclusion of the house, while the funeral is public. Joardar has shown that Bangladeshi people define ‘good death’ as that which takes place in the presence of loved ones (Joardar et al., 2014). In contrast to the notion of autonomy in Western culture, the collective or relational self plays a more prominent role in individual life in Bangladesh. The study also shows how the response to terminal illness and death depends on local worldviews which contain beliefs in rebirth or life after death as an integral part of life.

The practices and beliefs of providers of end of life care are also framed by culture. There is debate over whether and how the end of life care provider's culture in any given context can always be sensitive to the culture of the patient and his/her relatives, an issue sometimes framed as one of ‘cultural competence’ (Evans et al., 2012). The possible ‘clash of cultures’ becomes particularly problematic when the providers of palliative care in non-Western countries take guidelines developed in the Western context as their standard (Crippen, 2008). We also need to acknowledge that various social and cultural values of the wider society are played out in the practice of medical care itself (Zaman, 2004).

In this context of global disparity and variation in how palliative care might be both framed and practiced, it is now being argued that the quest for a global health system offering universal health coverage should include palliative care as a fundamental goal (WHPCA, 2016). WHO (2014) states that ‘Universal health coverage (UHC) means that all people receive the health services they need without suffering financial hardship when paying for them. The full spectrum of essential, quality health services should be covered including health promotion, prevention and treatment, rehabilitation and palliative care.’

Against this background, particular questions arise: what kind of future end of life care do we envision for developed and developing countries within the overall goal of providing universal health coverage? And how can different end of life care models interact with each other? We are using the term ‘developed’ and ‘developing’ countries here for convenience and are aware of the problems of such categorizations based on judging the development status of the countries. Our sensitivity to these distinctions should be revealed in our use of the following ideas about the ‘waiting room of history’.

### 1.2 ‘The waiting room of history’

The idea of ‘the waiting room of history’ has been introduced by Chakrabarty (2007) within postcolonial subaltern theory. Chakrabarty distinguishes between two types of history that have arisen with the spread of capitalism and the emergence of the modern world. ‘History 1’ is the story of capital, of how it makes or becomes itself. ‘History 2’, by contrast, is the history that does not belong to the life process of capital, and which may not be subsumed into the narrative of its progress; it lives in intimate and plural relationships, and this perspective allows us to make room for human diversity and the politics of belonging.

It is generally assumed that the process of modernity began in Europe. ‘History 1’ is therefore the history of Europe and the extension of European values throughout the world. According to this history, Europe is seen to be fully modern, while the rest of the world retains many pre-modern elements. Chakrabarty discusses how the classic liberal essays by John Stuart Mill ‘On Liberty’ and ‘On Representative Government’ both proclaimed self-rule as the highest form of government and yet argued against giving this to Indians or Africans. Chakrabarty writes:

‘According to Mill, Indians or Africans were *not yet* civilised enough to rule themselves. Some historical time of development and civilisation (colonial rule and education, to be precise) had to elapse before they could be considered prepared for such a task. Mill's historicist argument thus consigned Indians, Africans and other ‘rude’ nations to an imaginary waiting-room of history’ (Chakrabarty, 2007 p. 8).

In this schema, the mission of the rest of the world is to try to catch up with Europe or the West. For Chakrabarty, Europe is a particular mind-set associated with values that originate in the Enlightenment, but which are followed today in most parts of the modern globalised world. He argues that Enlightenment reasoning places non-European societies in a time that precedes that of Europe; a past in the present. Such societies therefore remain in the waiting room of history, aspiring to the future, that of Europe. Ahiska (2010) elaborates this point by stating that,

‘The ‘waiting room of history/civilization’ denotes a yearning for recognition from an imagined authority. But it also confides a story of self–depreciation projected from the imagined Western gaze, which has deemed local experience, as such, unworthy of knowledge and register’ (Ahiska, 2010:38).

To bring this idea of ‘the waiting room’ of history into the realm of palliative care we begin by drawing attention to two statements from two key figures in the field of palliative care.

### 1.3 Two statements

Dr. Jan Stjernswärd was appointed as Chief of Cancer to work at the WHO in 1980, where with colleagues from around the world he went on to play a key role in defining, promoting, and implementing palliative care in the global context. At the time of Stjernswärd's appointment, palliative care had not been recognised or defined by WHO and was not yet considered as a public health issue. Stjernswärd played a vital role in establishing the discourse of palliative care as a global health issue. Consider this key statement made by Stjernswärd in relation to these ambitions and found in the first edition of the *Oxford Textbook of Palliative Medicine*, published in 1993. Stjernswärd wrote the concluding chapter on the global perspective of palliative care, in which he states:

“Palliative medicine must from the beginning, encompass a global perspective, with the goal to reach the greatest number of people who can benefit from it, namely those in the developing countries. There cannot be one ideal future for the developed nations and another future for the developing nations. It is either one joint future or none”. (Stjernswärd, 1993, p.803).

In this telling statement, Stjernswärd raises two key points. On the one hand, he talks about the issue of inequity of palliative care provision between developed and developing countries. On the other, he raises the idea of a ‘common future’ for palliative care across the globe. This was to be an enduring vision. Twenty years later, in a personal memoir, Stjernswärd (2013) again emphasized the need for a ‘common global future’ for palliative care, stressing that it had been a constant theme in his work since the time of his appointment to WHO.

Although this notion is well meant, we are concerned about the underlying goal that it represents. We take the view that while the disparity in palliative care delivery between developed and developing countries is clearly evident, the concept of a ‘common future’ for end of life care for all countries is both challengeable and in need of clarification. It is increasingly assumed that a ‘good death’ and access to palliative care can be seen as human rights, and something that should be ‘common’ in both developed and developing countries, reaching the greatest number of people. But can there be a shared approach to achieving this? Is there a single understanding of what constitutes the good death or what ideal, effective, sustainable and appropriate palliative care looks like?

Consider, now and in contrast, a second statement from one of the pioneers of palliative care in India – Dr. Suresh Kumar, a physician, activist and notable leader of developments focussed on community strategies, and indeed sometime collaborator with Stjernswärd. In a 2012 interview Kumar states:

Despite attempts from various corners for more than three decades, globally, palliative care is accessible to only less than 8% of the needy today. It should be obvious to everybody by now that we are unlikely to achieve any meaningful coverage ever if we continue to take the conventional track. We need to be more innovative (Kumar, 2012).

Kumar is pointing at the same global disparity of palliative care to which Stjernswärd refers, but argues that the ‘conventional track’ – for which we read the approaches advocated by WHO - is not the answer. Abel et al. (2011) have explored how specialist palliative care, within hospices in particular, has historically led and set the standard and the conventional track for caring for patients at the end of life. Kumar advocates for a more innovative approach. For him this is to be found in a model of community participation in palliative care. Supporting this view Abel et al. (2011) point out three limitations of the conventional track of palliative care. First, the development of palliative care over the last 50 years in many countries has gone hand in hand with communities' loss of ownership of dying. There is now an expectation, particularly in the developed world, that when someone is dying, care will be given primarily by professional carers. Second, Western palliative care sees the dying person holistically, but crucially as an individual. The patient is perceived as an autonomous physical, emotional, social and spiritual being - dimensions that are all addressed by the palliative care team. In this context ‘social’ typically refers to the context of the nuclear family, so palliative care professionals tend to relate to a ‘patient-in-family’. Third, there is no research evidence on how—in the absence of any professional encouragement to do so—families mobilise their social networks at the end of life. It is not known therefore how formal services can complement naturally occurring support networks. Given this context, Abel and colleagues discuss how since the start of the twenty first century, the concept of ‘health promoting palliative care’ has gained interest and has advocated for the notion that care for terminally ill people should be returned to communities, albeit within which medical, nursing and other specialist services have an important place. Typically this alternative approach draws on the concept and theory of social capital, and the relevance of social connections, together with related norms and trust.

This is not to overlook the likelihood of wide variations, not only between individuals but also between communities, in which ethnicity, religion, social class, gender, personality, family and neighbourhood dynamics can all be influential (Graham and Clark, 2005). Abel and colleagues argue that because of the economic constraints on the development of expensive professional care and the potential for carer burnout, the focus should be upon supplementing a *service delivery* model with an alternative *community development* model (Abel et al., 2011).

### 1.4 Alternative palliative care models

Kellehear (1999) has outlined a health-promoting approach to end of life care through a series of actions by communities, governments, state institutions and social or medical care organisations that aim to improve health and wellbeing in the face of life-limiting illness. Building on this he has also proposed the idea of ‘compassionate communities’ (Kellehear, 2005) in which. care for one another at times of crisis and loss is not a task solely for health and social services, but rather is ‘everyone's responsibility’ (Kellehear, 2013). There are now examples throughout the world of community oriented end of life care in this vein. It is widely argued that perhaps the most refined version of the model is The Neighbourhood Networks in Palliative Care (NNPC) in Kerala, India, of which Suresh Kumar, is one of the founding members (Vijay and Kulkarni, 2012, Abel et al., 2011; Kumar and Palmed, 2007). The NNPC is an attempt to facilitate a sustainable, community-led service capable of providing palliative care to all those in need, with limited resource. The model begins from the perspective that chronic and incurable illnesses are social problems with medical components, rather than the commonly held converse view. It is based on the theory of primary health care outlined by the WHO in the *Declaration of* Alma Ata (1978). This states that primary health care is: ‘essential health care based on practical, scientifically sound and socially acceptable methods and technology made universally accessible to individuals and families in the community through their full participation and at a cost that the community and country can afford to maintain at every stage of their development in the spirit of self-reliance and self-determination’. Although few of the ideals of *Alma Ata* have been realized in the 30 years since its declaration, the Millennium Declaration and the Millennium Development goals set by the United Nations in 2000 re-opened the debate on primary healthcare and *Alma Ata* once again came to be championed as a method for achieving universal access to healthcare. The NNPC is an example of the revival of *Alma Ata* ideas and has in turn been acknowledged by WHO as a demonstration project (Stjernswärd, 2005). At the same time it raises an important question about the ways in which welfarist ideals are incorporated by the neo-liberal state and wherein vital areas of public welfare are ‘transformed from the discourse of a “right” to that of charity’ (Santosh, 2016).

There are scattered examples of community development public health approaches to palliative care in the richer countries like Singapore and Switzerland (Chan et al., 2014, Eychmüller and Benedetti, 2012). These examples make claim to being, ‘by and for’ the community, they draw on wider theoretical perspectives of social ecology, community action, empowerment and mobilisation. They should be distinguished from ‘community’ palliative care services provided by health professionals that are based in primary care settings, hospices or are delivered in people's homes. This also brings us to the distinction between ‘old’ and ‘new’ public health approaches. As Kellehear and Salnow (2012) point out, the ‘New Public Health’ is not a set of public health initiatives that add to the list of things that professionals do ‘TO’ communities, rather it is a new approach to health and social care and a collection of changes to enhance health and safety that can be done ‘WITH’ the community. In a systematic review, Sallnow et al. (2015) argue that issues of social isolation, carer support, personal and community capacity as well as wellbeing increasingly feature in policy documents for end of life care, that engaging communities is a way to meet these goals, and that positive impact can be demonstrated.

In general community development palliative care approaches appear to be gaining momentum and interest, albeit at a modest level. However, the rise in interest in these approaches has not been matched by a rise in the evidence supporting their use (Sallnow et al., 2015). Perhaps for these reasons, there is a dearth of discussion about the role, feasibility and value of these alternative models in debates about the global future of end of life care. It would seem that as yet, we are unable to find a ‘common’ answer to the question about the global future of end of life care. Should care of people with life limiting illnesses and those approaching death be a ‘specialised service’ or the ‘business of everyone’? Stjernswärd's assertion that “there should be either one common future for palliative care or none”, commits to a linear developmental vision of the future - one that is loaded with universalising tendencies.

Similar notions have appeared in the narrative of other global health issues. For example, Titchkosky and Aubrecht (2015) in their study of the mental health programme of the WHO show how it provides a singular, even totalizing conception of human suffering and how the WHO mental health treatment protocol can be read as a colonizing force in post-colonial times. Similarly McPhail-Bell, Fredericks and Brough (2013) show that the narrative informing the development of the renowned Ottawa Charter for health promotion strongly reflected Western/colonizer-centric worldviews, and actively silenced the possibility of countervailing indigenous and developing country voices.

### 1.5 Common or multiple futures?

Our argument therefore centres on the question of whether we should we aim for a common global future for end of life care, or look for multiple possibilities. We suggest that there are risks in transferring the idea of a policy for ‘one joint future’ of end of life care from the developed to the developing nations. The ‘quality of death’ ranking by *The Economist* is a remarkable illustration of the ‘the waiting room of history’ The ranking shows mostly the Western developed countries, following the conventional specialist palliative care path with the UK leading the table (The Economist, 2015). There is an implied assumption in this ranking that those at the bottom, most of which are developing countries, should aspire to the level of quality of death of the top ranking countries. There is a paradox here. Recent evidence shows that even within the UK, a pioneering country in the palliative care field and head of the Quality of Death Index, there is much concern and a measure of disagreement about how well palliative and end of life care are being delivered ‘at scale’ - seen in the furore over the Liverpool Care Pathway (Sleeman, 2014) and in poor reports on end of life care from the Ombudsman and the Care Quality Commission (Dyer, 2015).

We also need to recognise that the medical landscape in which palliative care operates, particularly in the contemporary Western world, has become much more complex than it was when palliative care was first developing as a specialty. As a result, new debates have emerged that remain unresolved. Commentators have argued that a process of medicalization is taking place within palliative care, overriding its early, more holistic intentions. The issue has become more complicated with the distinction between cancer and non-cancer palliative care, particularly when cancer in many Western countries has transitioned from a terminal to a chronic disease. Palliative care has therefore come to focus on patients with complex multiple problems, who need care over a long period of time. These shifts pose a challenge to the conceptual and practical aspects of palliative care. Furthermore, new medical, epidemiological and demographic developments have generated debates and arguments in relation to the ethical, moral and legal aspects of assisted dying and euthanasia, particularly in affluent countries. There is ongoing and unsettled debate in many Western countries over whether to allow persons a measure of control over the manner and timing of their death. The question therefore must be posed as to whether it is responsible to ‘roll out’ models of palliative care intervention developed in the West across settings with immense social, political and cultural differences.

The *Economist* index provides minimum space for the alternative community development models of palliative care we have highlighted here. Indeed, ‘community engagement’ is given the lowest weighting among five indicators in the ranking. The ranking illustrates clearly the notion of the ‘waiting room of history’: the bottom ranking countries are deemed ‘not yet’ in a position to attain ‘quality of death’ and must pass through a period of historical time for further development, before their place in the index can improve.

By contrast, we would argue that the ‘good death’ contains two conceptual components: value and logistics. By ‘value’ we are referring to one's ‘judgement’ about a good death. In turn ‘logistics’ denotes the arrangements to achieve the stated ‘value’. In the current narratives of the good death, the value includes a dignified, pain free and controlled process (Hart et al., 1998, Cottrell and Duggleby, 2016) and for that the required logistics are well equipped hospice and palliative care facilities, trained professionals and opioid availability, which have been ensured to a greater or lesser extent in the global North. Nevertheless as we have shown, the ‘good death’ does not have a singular global narrative. If we consider the alternative narratives of good death where dignity, autonomy even a pain free death are not the priorities, it then might be possible to achieve a good death without the prescribed conventional ‘logistics’. A good quality death could probably be achieved by making the most of community assets and capacity. For example, Abel and Kellehear (2016) suggest that a good quality death can be achieved through ‘network development’, ‘development of supportive communities of volunteers’ and ‘creation of compassionate policies’. If we consider these as the tools and arrangements, in other words the ‘logistics’ to achieve a good death, many countries do not need to sit in the imaginary ‘waiting room’ before they can develop an appropriate palliative care service model. NNPC, Kerala for example demonstrates how with the support of the specialists, ‘community assets’ can be the main ‘logistics’ to achieve a good quality death for large numbers.

### 1.6 Transfer or translation?

Chakrabarty (2007) raises major concerns about the uncritical transfer of ideas, practices and narrative from one context to another. He argues in the context of political modernity that we should stop looking at history as a developmental process that pays attention solely to the *transfer* of the modernity of Europe to other places. Instead, we need to look at a history (or histories) of the *translation* of modernity from Europe to other parts of the world through the multiple relationships that exist between them. This would allow us to understand the plurality of past and present and at the same time to anticipate plural futures.

Should we anticipate a common future in how we deal with the annual 15 million deaths in developed countries and the 40 million deaths in the developing countries? Should we aim for institutionalised, specialised palliative care services or community-led, generalist services that build on local social capital and the skills and capacities of the people? Should we focus on the transfer of palliative care narratives, assumptions, policies and practices from developed to developing countries, or should our emphasis be on the translation of these things in both directions?

We can relate this argument with the debate going on about another global issue, namely climate change. Within the climate change domain there is the principle of ‘common but differentiated responsibility’ (UN, 2002). The principle recognises historical differences in the contributions of developed and developing states to global environmental problems, and differences in their respective economic and technical capacity to tackle these problems. Despite their common responsibilities, important differences exist between the stated implications for developed and developing countries. For example, differentiated responsibility translates into differentiated environmental standards set on the basis of a range of factors, including special needs and circumstances, future economic development of countries, and historic contributions to the creation of an environmental problem.

Despite the fact that dying is a common human experience, we argue for more plurality in how it should be addressed. If we adopt a truly global perspective we will need to accept the plurality of past and present local problems and issues related to end of life care, as well as the plural possibilities of how they might be overcome. In that case, we would not aim to universalise or privilege one particular future for end of life care, globally. Instead of homogenising end of life interventions, we would seek to be open to multiple futures for the care of the dying. We also need to take into account that there are within-country variations and diversity in end of life care provision. It is therefore not only the developed and developing country divide that must be targeted, but also the unwelcome variation and inequity that exists *within* jurisdictions and communities.

We therefore recommend the following to deal with the future challenges of end of life care in the global context. It is important to identify common denominators of end of life care around the world and work from there to develop culturally and locally appropriate provision of palliative care. The aim should be not to privilege one particular future for end of life care globally but to seek a *suite of solutions*. In planning and developing palliative care policy it is crucial to explicitly conceptualize the country specific ‘value-logistic’ dimensions of ‘good death’ that we propose in this paper. It is also important to conduct more international comparative research on end of life interventions, using culturally and historically informed methods. Finally, it seems likely that the future of effective, culturally appropriate and sustainable approaches will depend on the identification of ‘the particular’ within ‘the universal’ at the end of life.

## Ethical approval

This paper did not involve data collection from human subjects, so ethical approval is not required.
